# Supplementary material for: Chemical diversity in a metal–organic framework revealed by fluorescence lifetime imaging
Source: Nat Commun. 2018 Apr 25;9:1647. doi: 10.1038/s41467-018-04050-w (PMC5916894; doi:10.1038/s41467-018-04050-w)
Supplement: Supplementary file 1 — Supplementary Information [file 41467_2018_4050_MOESM1_ESM.pdf]

Chemical Diversity in a Metal-Organic Framework Revealed by Fluorescence Lifetime Imaging

Waldemar Schrimpf *et. al.*

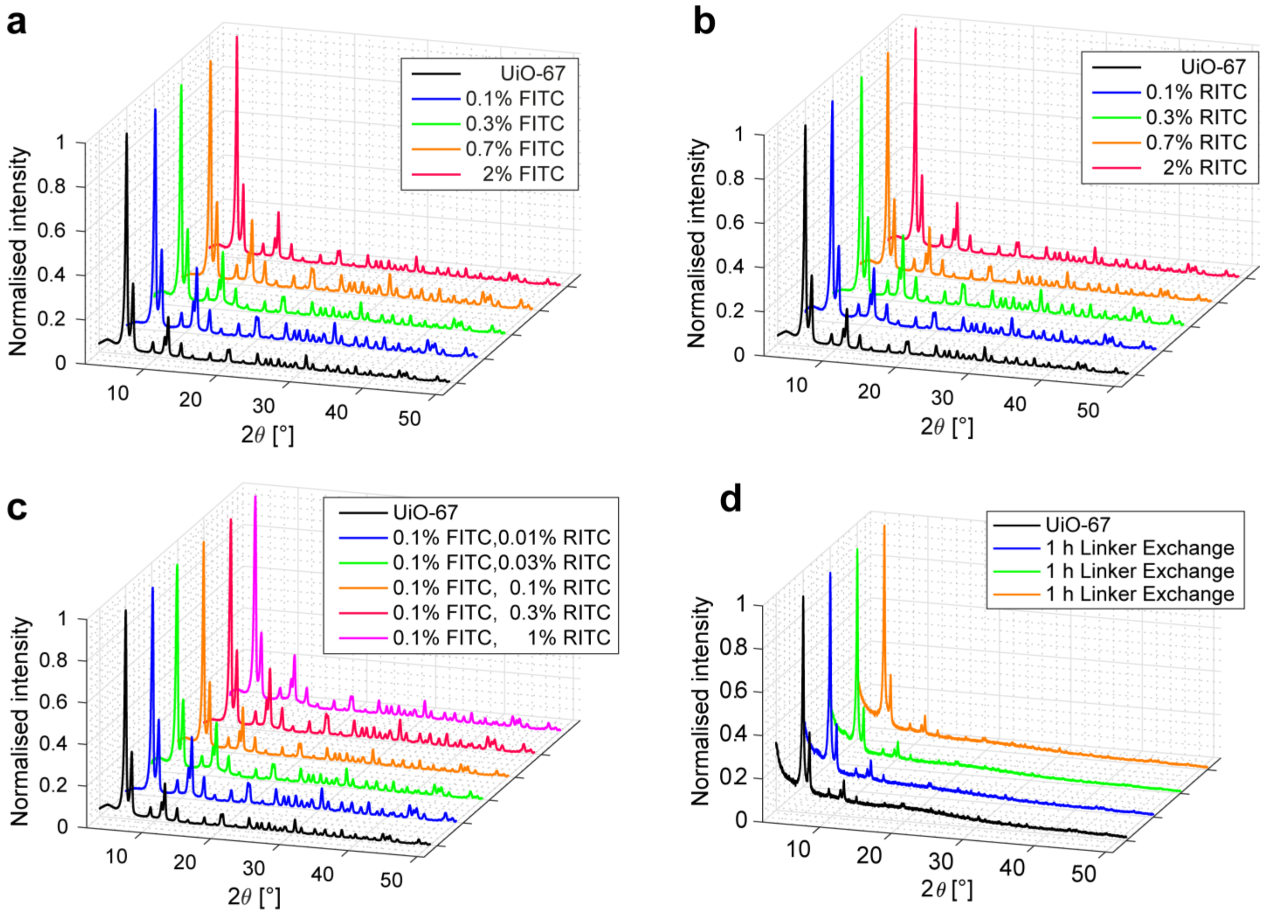

Supplementary Figure 1: XRD patterns of small crystal UiO-67 samples with varying amounts of dye-modified linkers. a) *De novo* functionalization with FITC modified linker. b) *De novo* functionalization with RITC modified linker. c) *De novo* functionalization with both FITC and RITC modified linkers. d) Samples functionalized with FITC modified linker using linker exchange.

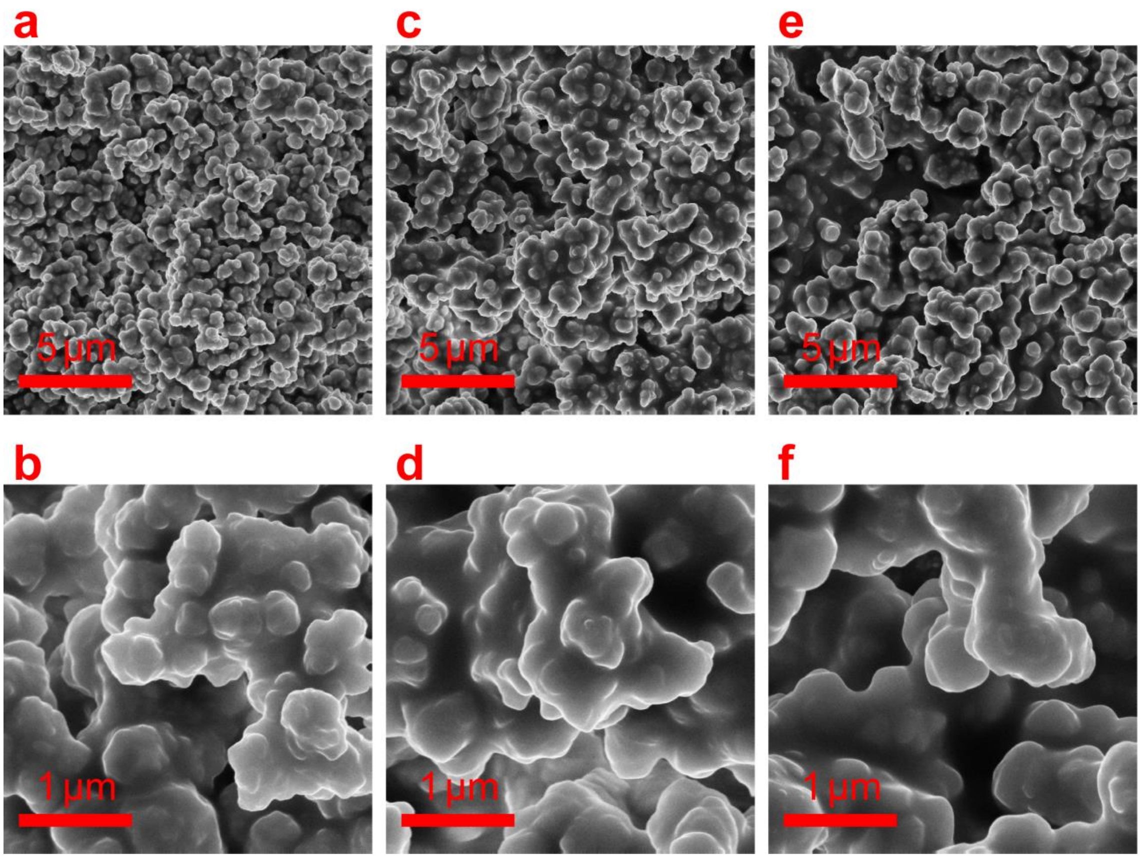

Supplementary Figure 2: SEM images of small crystal UiO-67 samples with 0% (a and b), 0.1% (c and d), and 2% FITC modified linker (e and f) used during synthesis.

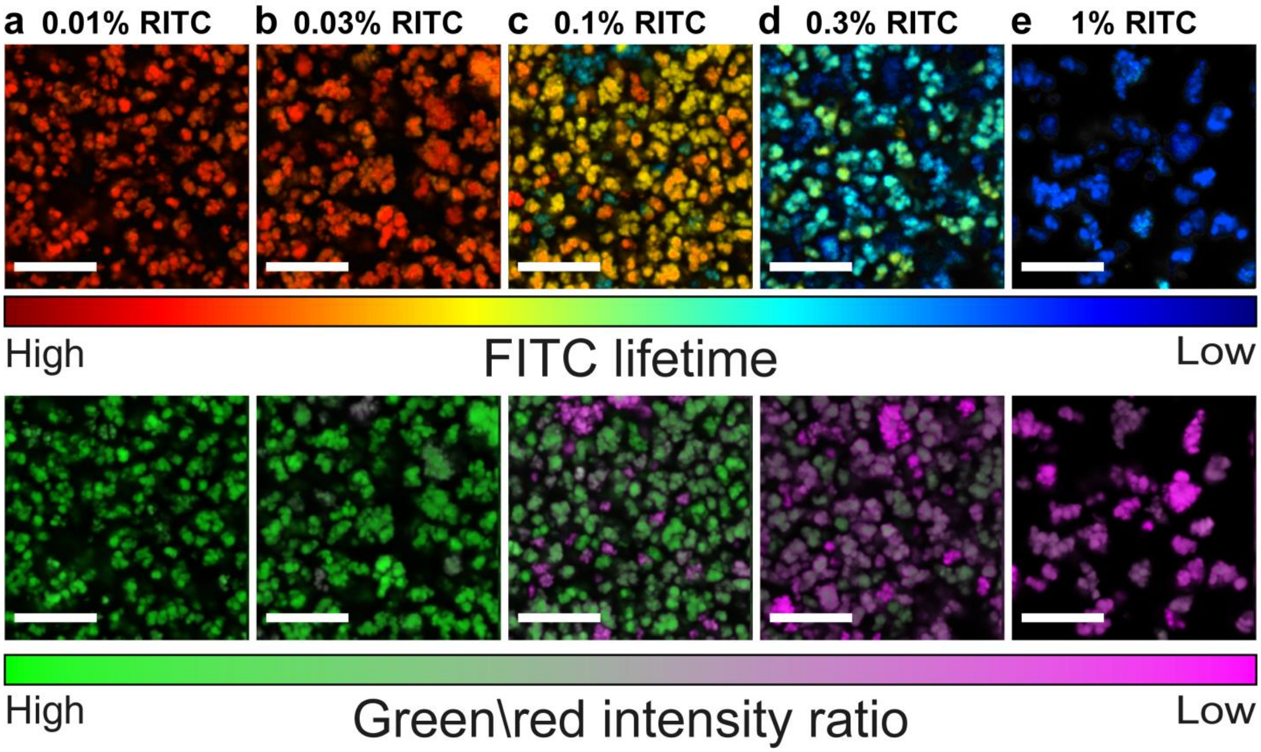

Supplementary Figure 3: UiO-67 samples with both FITC and RITC labelled linkers. The input FITC fraction for all samples is 0.1%, while the RITC content increases from left to right starting at 0.01% (a), 0.03% (b), 0.1% (c), 0.3% (d), and 1% (e). The upper images are identical to the ones shown in Figure 2. The lower images display the intensity ratio between the green (FITC) and the red (RITC) channels after excitation with a 475 nm laser. To optimize the contrast, the green signal was increased by a factor of 5 compared to the red channel.

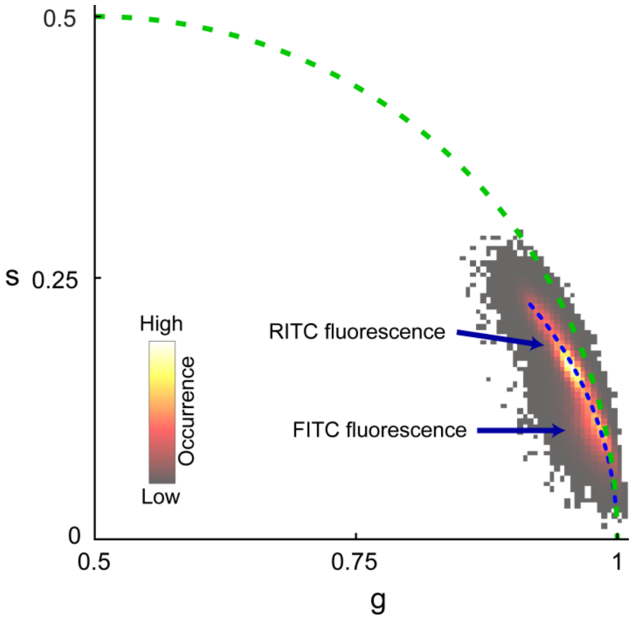

Supplementary Figure 4: A phasor histogram of the FITC and RITC (both after 475 nm and 565 nm excitation) lifetimes of the 0.1% FITC and 1% RITC *de novo* modified UiO-67 sample shown in Figure 1 h-k. The dotted blue line is used for the colour coding in Figure 1 h-k.

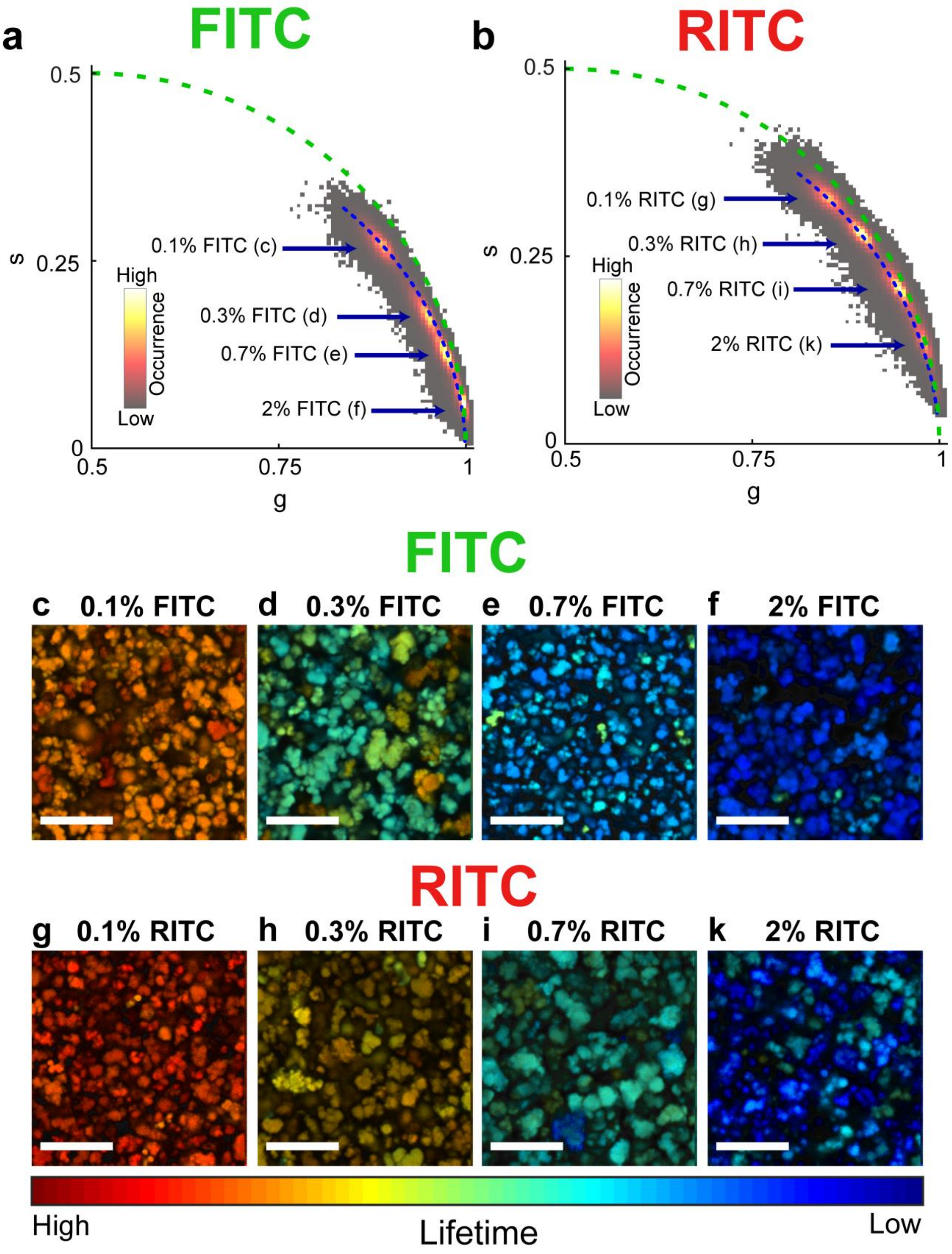

Supplementary Figure 5: Phasor analysis of *de novo* UiO-67 samples with either FITC or RITC labelled linkers. a-b) Phasor plots of the FITC (a) and RITC (b) fluorescence of the full images shown in c-k. The blue arrows indicate the average phasor positions of the different samples. c-f) Lifetime images of FITC fluorescence of UiO-67 samples with 0.1% (c), 0.3% (d), 0.7% (e), 2% (f) input FITC linker fraction. c-f) Lifetime images of RITC fluorescence of UiO-67 samples with 0.1% (c), 0.3% (d), 0.7% (e), and 2% (f) input RITC linker fraction. The scale bar for all images is 10 μm.

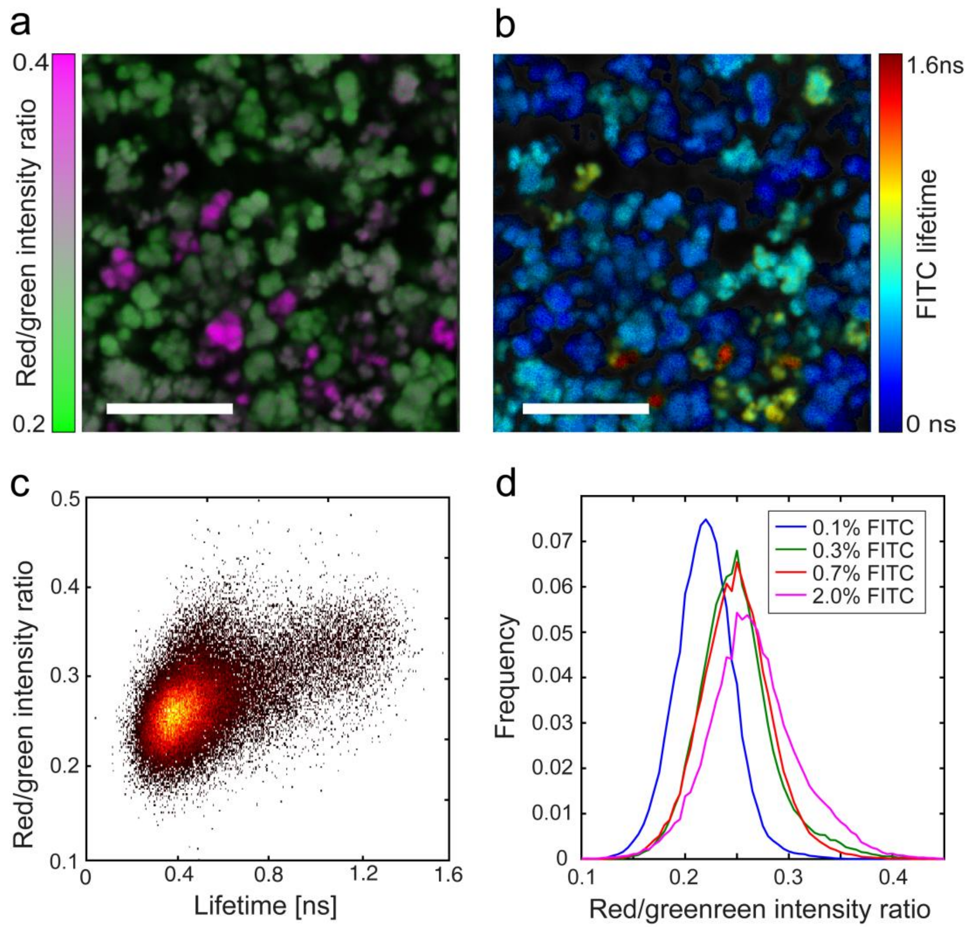

Supplementary Figure 6: Spectral shift of UiO-67 samples *de novo* functionalized with FITC modified linkers. a) An intensity ratio image showing the spectral shift of FITC fluorescence for individual pixels with 2% input FITC fraction. The spectrum is indicated by the ratio between the red (570-620 nm) and the green (500-540 nm) detection channels. b) Fluorescence lifetime image of the same region as shown in a. c) Pixelwise 2D histogram of the red/green intensity ratio vs. the lifetime of *de novo* functionalized sample with 2% input FITC fraction, showing a correlation between the spectral shift and the fluorescence lifetime. d) Distribution of red/green intensity ratio of individual pixels for different amounts of input FITC fraction. Based on the free FITC spectrum, the difference between the 0.1% and the 2% FITC samples correspond to a red-shift of 3-6 nm. The scale bar in the images is 10 μm.

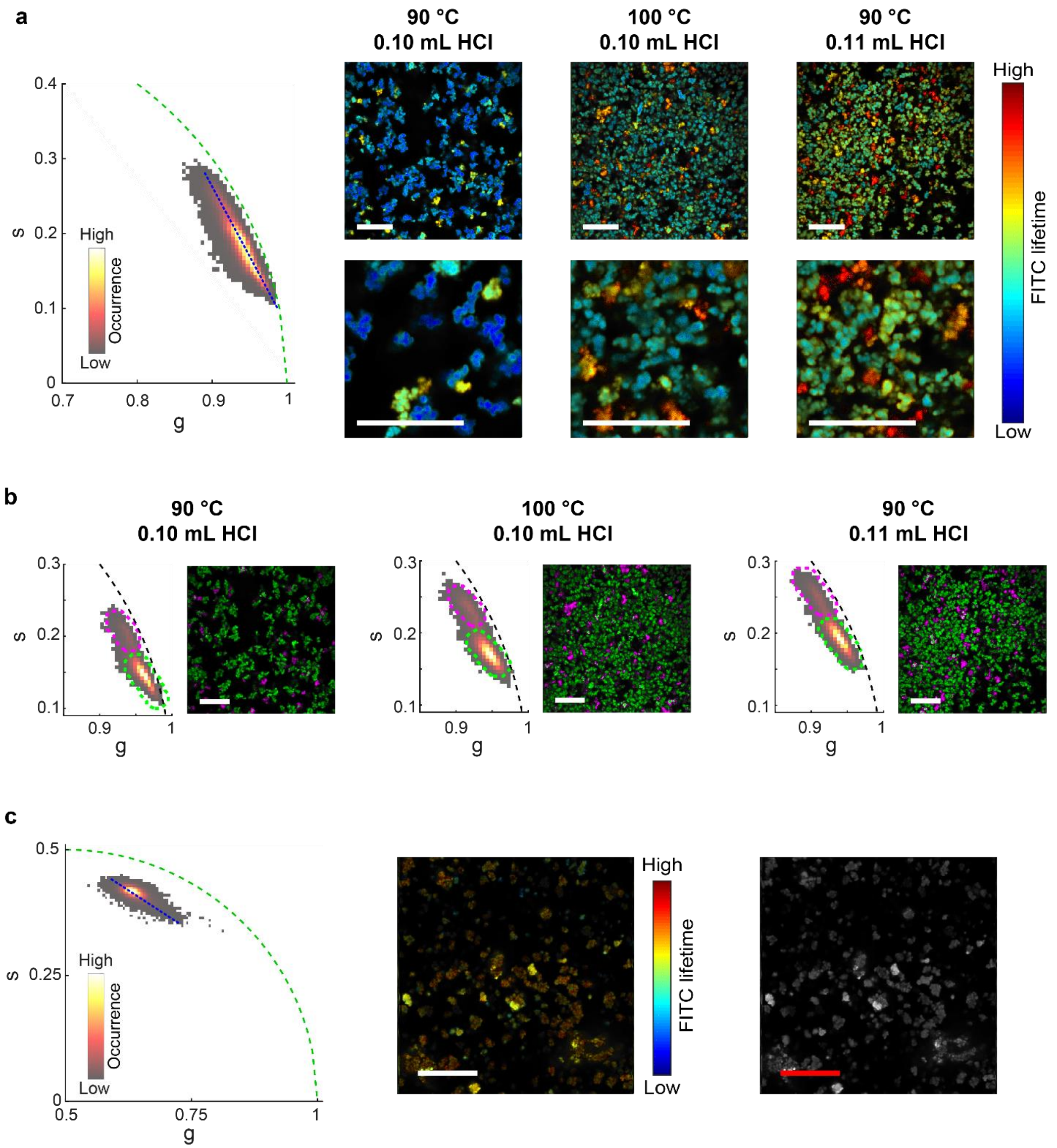

Supplementary Figure 7: *De novo* samples at different conditions. a) The phasor plot and images of FITC fluorescence of 0.7% FITC *de novo* samples synthesized with standard conditions (left), at elevated temperature (middle), and higher HCl concentration (right). The blue dotted line is used to colour-code the fluorescence lifetime in the images. b) Separate phasor plots and FLIM images of the samples shown in a. The green and magenta ellipses indicate the two distinct particle populations and are used to colour-code the corresponding particles in the images. c) The phasor plot (left), FLIM image (center) and intensity image (right) of UiO-67 auto-luminescence of a sample without added fluorophores. The blue dotted line is used to colour-code the fluorescence lifetime in the FLIM image.

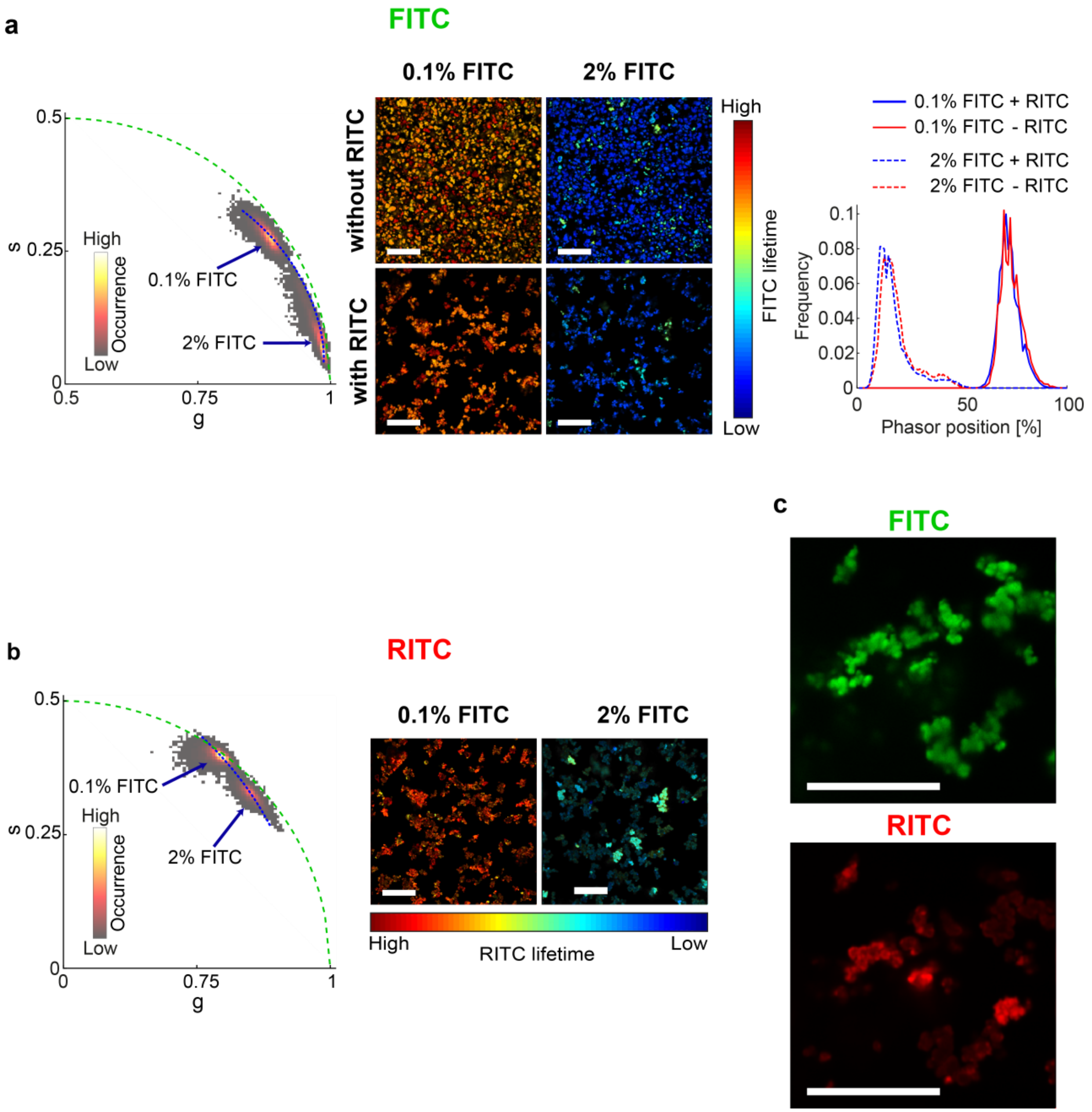

Supplementary Figure 8: Phasor analysis of UiO-67 samples functionalized *de novo* with FITC labelled linkers and post-synthetically with RITC. a) Phasor histogram (left), FLIM images (centre) and phasor distribution (right) of the FITC fluorescence of the 0.1% (left images and solid lines) and 2% (right images, dotted lines) FITC *de novo* samples before (upper images, bred lines) and after (lower images, blue lines) treatment with RITC. The colour-code of the images and the phasor distribution in the line plot represent the position along the blue dotted line in the phasor histogram. b) Phasor plot and FLIM images of the RITC fluorescence of the 0.1% (left image) and 2% (right image) FITC *de novo* samples after treatment with RITC. c) Intensity images of the FITC (left, green) and RITC (right, red) fluorescence for the same region. All scale bars are 20 µm.

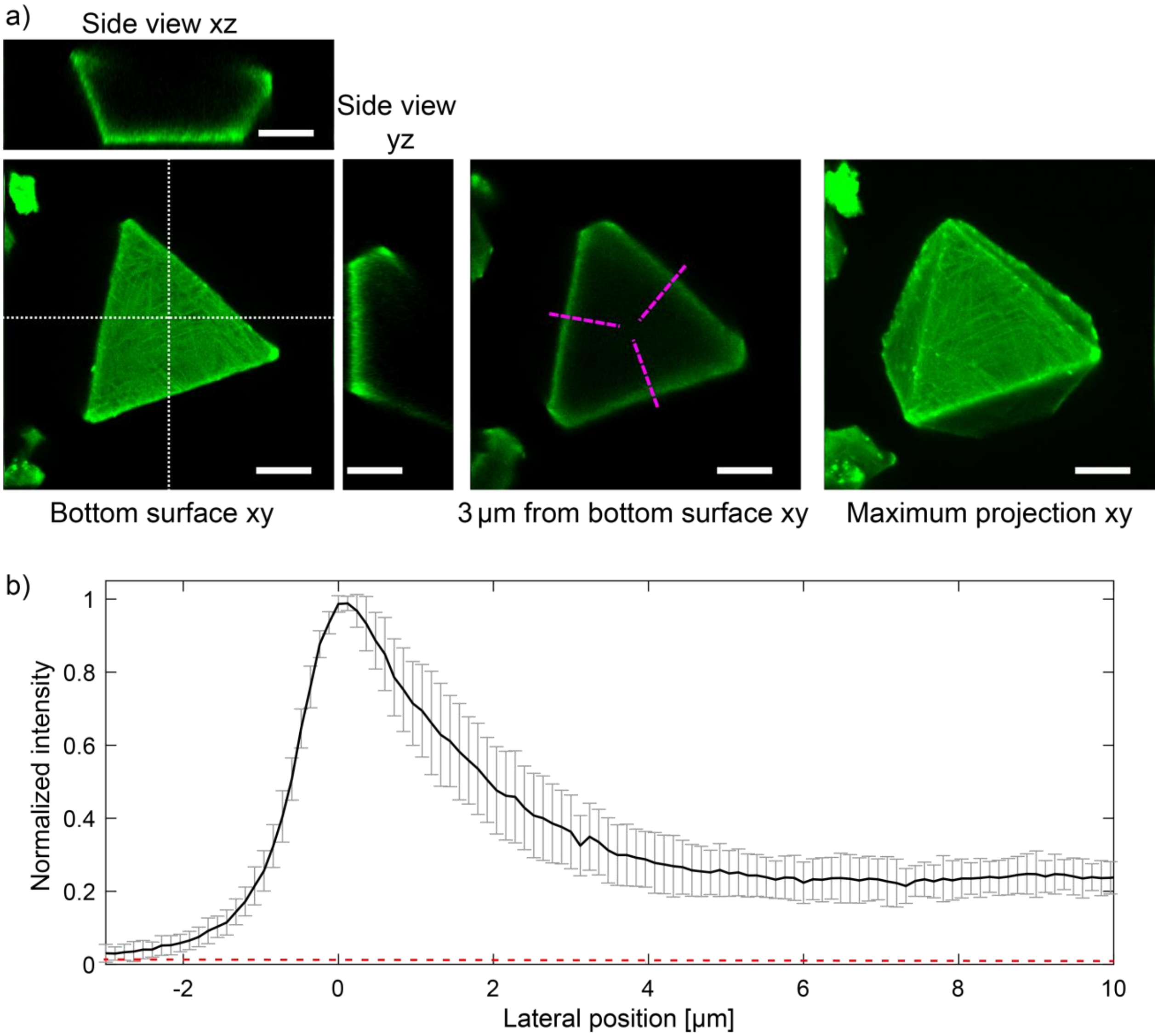

Supplementary Figure 9: Fluorescence intensity distribution in a large UiO-67 crystal functionalized with linker exchange. a) Z-Scan fluorescence intensity images showing a xy slice at the bottom of the crystal (left), 3 µm up from the bottom (centre) and a xy maximum projection image for visualizing the octahedral 3D structure. The side views show the horizontal (xz) and vertical (yz) slices through the centre of the xy images highlighted by the white dotted lines. The scale bar is 10 µm. b) Intensity profile perpendicular to the three surfaces of the xy plane 3 µm from the bottom surface, indicated by the three dotted magenta lines in panel a. The black line shows the average and the grey error bars indicate the standard deviation of the three sides. The red dotted line indicates the intensity expected from the auto-luminescence and the background (measured outside of the crystals) and corresponds to approximately 1% of the peak intensity.

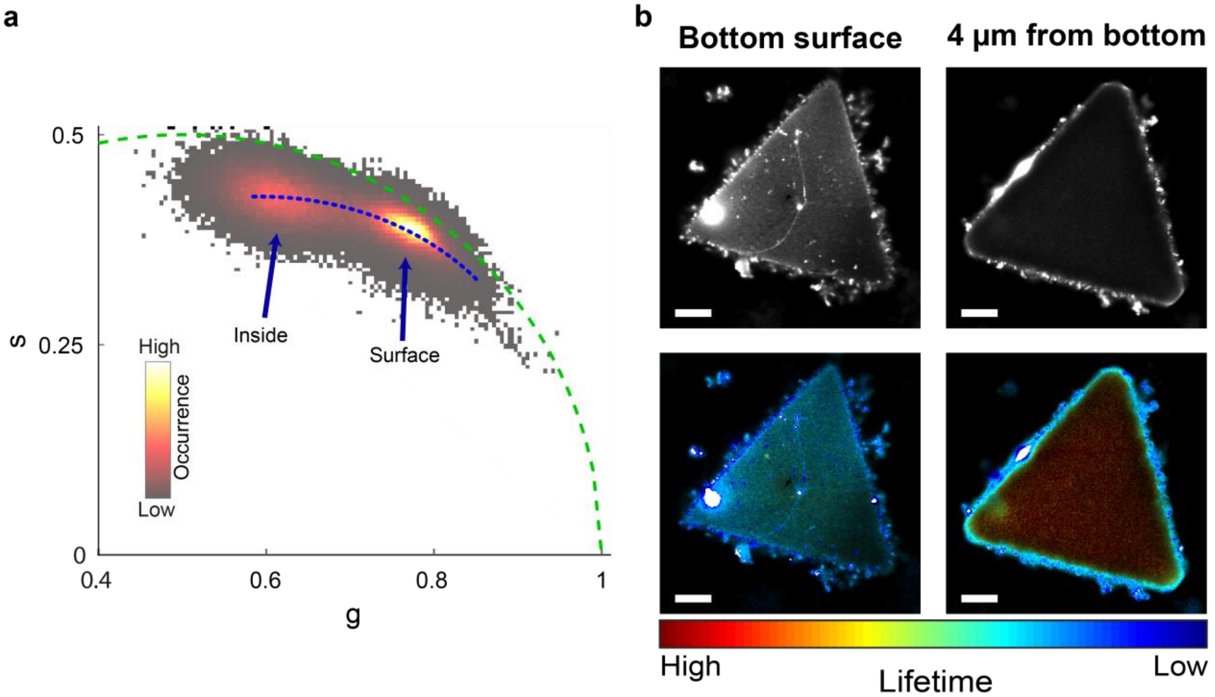

Supplementary Figure 10: Auto-luminescence of large UiO-67 crystal. a) The phasor plot of the auto-luminescence of a large UiO-67 crystal at the bottom plane (surface) and 4 µm from the bottom (inside). b) Intensity (upper images) and FLIM (lower images) images of the auto-luminescence at the bottom surface (left images) and 4 µm from the bottom (right images). The FLIM images were color-coded according to the phasor position along the blue dotted line in a) using the plotted color-table. The scale bar is 10 µm.

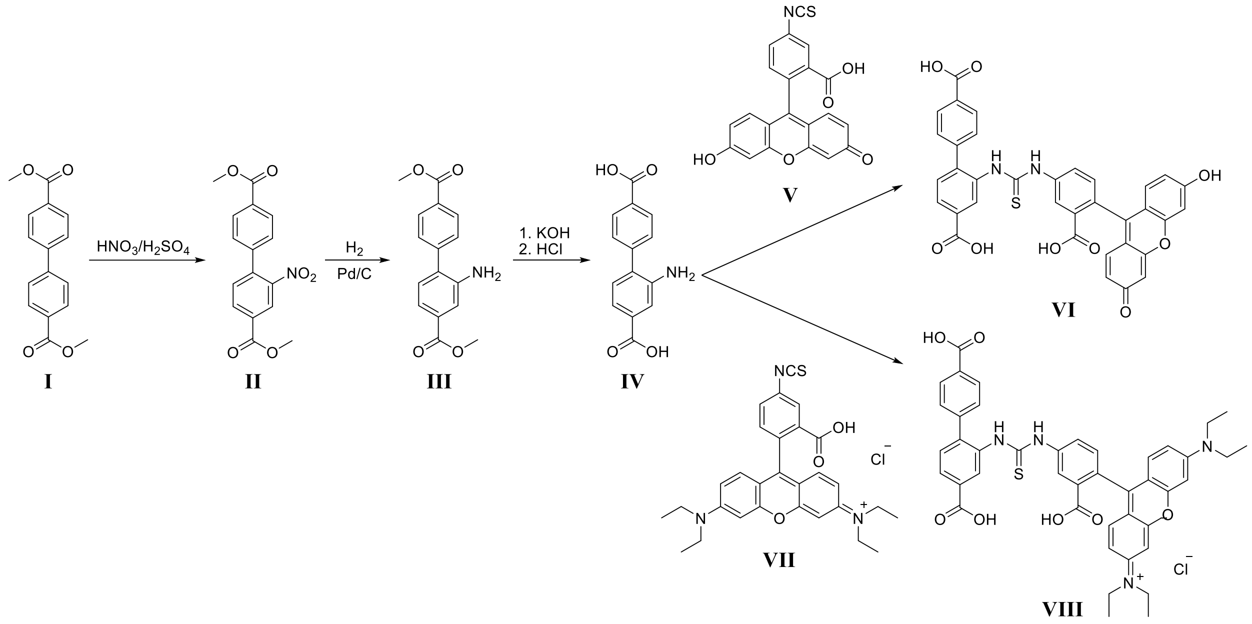

Supplementary Figure 11: A schematic of the synthetic approach used to generate dye-functionalized linkers. For details, see the *Synthesis of dye-functionalized linkers* section in the SI.

Supplementary Table 1: Dye incorporation and fluorescence lifetimes in FITC and RITC functionalized UiO-67 samples.

Sample	FITC				RITC			
	Conc. [ppm]	Incorp. Efficiency	Nearest Neighbour Distance [Å]	τ[ns]	Conc. [ppm]	Incorp. Efficiency	Nearest Neighbour Distance [Å]	τ[ns]
***de novo* functionalization**								
0.1% FITC	256	25.6	81.0	2.28±0.15	-	-	-	-
0.3% FITC	808	26.9	55.2	1.57±0.20	-	-	-	-
0.7% FITC	1624	23.2	43.8	1.09±0.17	-	-	-	-
2% FITC	3511	17.6	33.8	0.55±0.32	-	-	-	-
0.1% RITC	-	-	-	-	38	3.8	153	2.67±0.16
0.3% RITC	-	-	-	-	89	3.0	115	2.17±0.14
0.7% RITC	-	-	-	-	220	3.1	85.2	1.54±0.16
2% RITC	-	-	-	-	410	2.1	69.2	0.95±0.31
0.1% FITC, 0.01% RITC	212	21.2	86.3	2.06±0.18	7	7.0	269	-
0.1% FITC, 0.03% RITC	264	26.4	80.2	2.03±0.15	21	7.0	186	-
0.1% FITC, 0.1% RITC	238	23.8	83.0	1.70±0.17	64	6.4	129	-
0.1% FITC, 0.3% RITC	212	21.2	86.3	1.20±0.18	134	4.5	102	-
0.1% FITC, 1% RITC	212	21.2	86.3	0.83±0.26	529	5.3	63.6	-
**linker exchange functionalization**								
Small crystals, 1 h @ 65 °C	8188	-	25.5	2.11±0.23	-	-	-	-
Small crystals, 6 h @ 65 °C	7212	-	26.6	1.76±0.21	-	-	-	-
Small crystals, 24 h @ 65 °C	9561	-	24.2	1.40±0.15	-	-	-	-
Large crystals, surface	-	-	-	2.19±0.17	-	-	-	-
Large crystals, inside	-	-	-	2.73±0.19	-	-	-	-

Supplementary Table 2: BET surface areas and pore sizes of pure and dye *de novo* functionalized UiO-67 samples.

Sample	Octahedral Pore Size [Å]	Tetrahedral Pore Size [Å]	Surface Area[m²/g]
UiO-67	17.5	10.5	-
0.1% FITC	17.3	10.5	2796
0.3% FITC	17.6	10.5	2195
0.7% FITC	17.7	10.6	2705
2% FITC	17.7	10.5	2451
0.1% RITC	18.1	10.5	2765
0.3% RITC	17.6	10.6	2738
0.7% RITC	17.9	10.4	2567
2% RITC	17.6	10.6	2597
0.1% FITC, 0.01% RITC	16.9	10.7	2674
0.1% FITC, 0.03% RITC	17.4	10.5	2555
0.1% FITC, 0.1% RITC	17.4	10.5	2600
0.1% FITC, 0.3% RITC	17.6	10.6	2648
0.1% FITC, 0.3% RITC	17.4	10.5	2568

Supplementary Table 3: Amount of linkers used for *de novo* functionalization.

Sample	H_2BPDC [mg]	FITC-H_2BPDC [mg]	RITC-H_2BPDC [mg]
UiO-67	18.8	-	-
UiO-67, 0.1% FITC	18.8	0.0502	-
UiO-67, 0.3% FITC	18.7	0.1506	-
UiO-67, 0.7% FITC	18.7	0.3514	-
UiO-67, 2% FITC	18.4	1.004	-
UiO-67, 0.1% RITC	18.8	-	0.0638
UiO-67, 0.3% RITC	18.7	-	0.1914
UiO-67, 0.7% RITC	18.7	-	0.4466
UiO-67, 2% RITC	18.4	-	1.276
UiO-67, 0.1% FITC, 0.01% RITC	18.8	0.0502	0.00638
UiO-67, 0.1% FITC, 0.03% RITC	18.8	0.0502	0.01914
UiO-67, 0.1% FITC, 0.1% RITC	18.5	0.0502	0.0638
UiO-67, 0.1% FITC, 0.3% RITC	18.7	0.0502	0.1914
UiO-67, 0.1% FITC, 1% RITC	18.3	0.0502	0.638
